# Stool multi-omics for the study of host–microbe interactions in inflammatory bowel disease

**DOI:** 10.1080/19490976.2022.2154092

**Published:** 2022-12-11

**Authors:** Consuelo Sauceda, Charlie Bayne, Khadijeh Sudqi, Antonio Gonzalez, Parambir S. Dulai, Rob Knight, David J. Gonzalez, Carlos G. Gonzalez

**Affiliations:** a Department of Pharmacology, University of California San Diego, La Jolla, CA, USA; b Skaggs School of Pharmacy, University of California San Diego, La Jolla, CA, USA; c Center for Microbiome Innovation, University of California San Diego, La Jolla, CA, USA; d Department of Pediatrics, University of California San Diego, La Jolla, CA, USA; e Department of Bioengineering, University of California San Diego, La Jolla, CA, USA; f Department of Computer Science and Engineering, University of California San Diego, La Jolla, CA, USA; g Division of Gastroenterology and Hepatology, Northwestern University, Chicago, IL, USA

**Keywords:** Inflammatory bowel disease (IBD), Multi-omics, gut microbes, diet, precision medicine, metagenomics, metaproteomics, metabolomics

## Abstract

Inflammatory Bowel Disease (IBD) is a chronic immune-mediated inflammatory disease of the gastrointestinal tract that is a growing public burden. Gut microbes and their interactions with hosts play a crucial role in disease pathogenesis and progression. These interactions are complex, spanning multiple physiological systems and data types, making comprehensive disease assessment difficult, and often overwhelming single-omic capabilities. Stool-based multi-omics is a promising approach for characterizing host-gut microbiome interactions using deep integration of technologies such as 16S rRNA sequencing, shotgun metagenomics, meta-transcriptomics, metabolomics, and metaproteomics. The wealth of information generated through multi-omic studies is poised to usher in advancements in IBD research and precision medicine. This review highlights historical and recent findings from stool-based muti-omic studies that have contributed to unraveling IBD’s complexity. Finally, we discuss common pitfalls, issues, and limitations, and how future pipelines should address them to standardize multi-omics in IBD research and beyond.

## Introduction

Inflammatory bowel diseases (IBD) are chronic immune-mediated inflammatory diseases of the gastrointestinal tract. The two major IBD subtypes are ulcerative colitis (UC) and Crohn’s disease (CD). UC and CD are often sub-classified based on phenotypic presentations according to clinical and endoscopic severity, disease location (foregut, ileal, extent of colonic involvement, perianal, or combinations of these in the case of CD), and presence or absence of strictures and penetrating complications. The complex influence of host genetics, sex, ancestry, and environmental factors including diet and gut-microbial composition in IBD has overwhelmed the utility of current phenotypic classification efforts, which rely on endoscopies, nonspecific markers of inflammation (C-reactive protein or fecal calprotection) and symptom-based assessments.^1,2^ Furthermore, current standards for diagnosis and assessment of treatment response often require multiple invasive screenings, which is taxing on patients and clinicians. This inability to accurately capture heterogeneity in disease mechanisms driving phenotypic presentation has hampered accurate diagnosis and treatment efforts and increased economic burdens.^3–6^

Given this evidence, it is unsurprising that current disease classification metrics and disease severity monitoring strategies that guide IBD treatment have been increasingly questioned.^7–10^ As such, the field needs diagnostics and monitoring tools that are low cost, minimally invasive, and accurately capture IBD’s multifactorial complexity. Such tools would likely be derived from the molecular underpinnings that distinguish disease subtypes with greater granularity. Unfortunately, current work defining these subtypes and their molecular profiles is limited but possesses enormous potential for driving precision medicine in IBD.

While clinicians have long suspected that a myriad of heterogeneous IBD phenotypes exist, a comprehensive understanding of these phenotypes was hindered by the lack of profiling technologies with adequate resolution to capture the observed heterogeneity. Since then, genome-wide association screens have confirmed genetic variants associated with T-cell phenotype imbalances, increased neutrophil activity (*nod1/2*), and antigen presentation (e.g. HLA variants) contribute to disease etiology.^11–14^ Additional efforts identified altered host antimicrobial responses and perturbations to intestinal epithelial integrity as key mediators of disease activity, further highlighting microbial contribution to IBD, yet still leaving researchers searching for cohesive mechanisms explaining these observations.

However, the rise of ever-more powerful -omic technologies has refined our understanding of IBD as a complex and dynamic disease composed of many possible interactions between host and gut microbes.^14^ Supporting this connection, recent IBD patient profiling efforts suggest that host immune tone heavily influences the gut-microbe composition,^13,15^ resulting in increased proportions of the phylum *Bacteroidetes* and decreased proportions of *Firmicutes* relative to healthy populations.^16–19^ These findings suggested that dysbiotic microbial composition contributes to IBD.^19^ Since then, research on host-microbe crosstalk has provided evidence supporting pathologic roles for specific microbes, with effects ranging from barrier disruption to influencing immune cell repertoire. Given its ability to influence microbial growth, dietary intake has also now been implicated as critical to IBD pathology and treatment.^20–22^ These revelations led to metabolic studies suggesting microbial products such as short-chain fatty acids influence can modulate host immune systems.^23^ Despite these gains in knowledge, we are only beginning to understand the molecular network of factors influencing IBD heterogeneity, which significantly complicates progress toward precision medicine goals.

**Figure 1.**
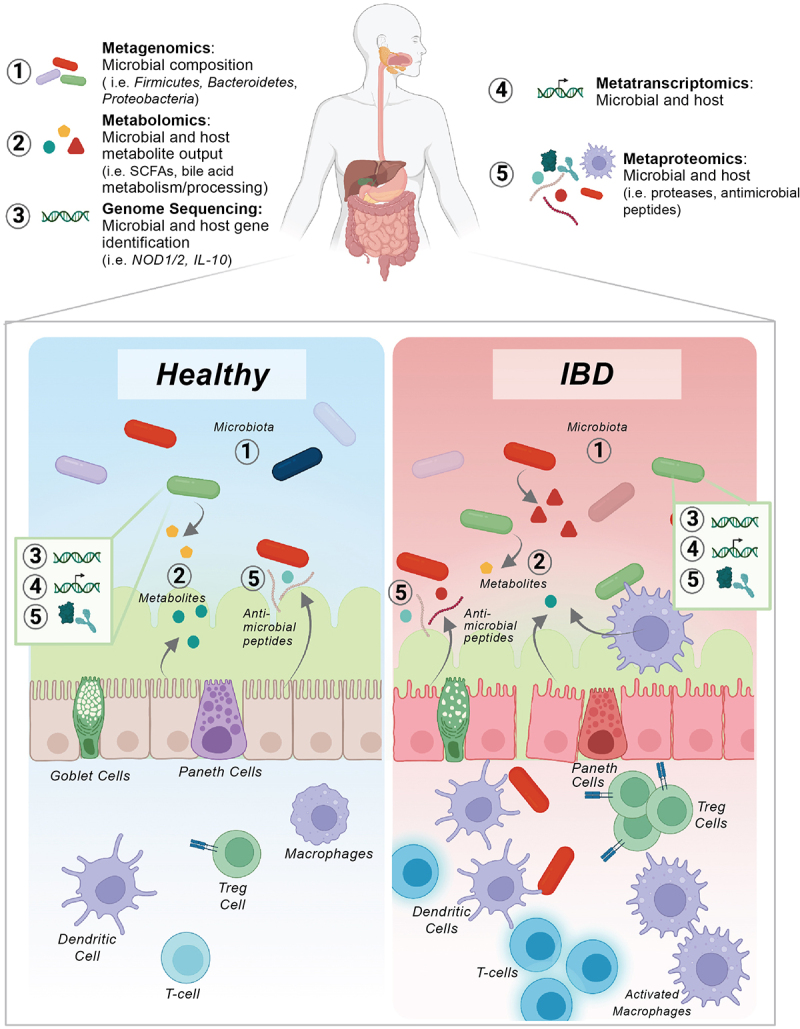
Microbe-host multi-omic targets in healthy and diseased intestinal microenvironment. Visual representation of the integration potential of multi-omics. 1) Advancements in metagenomics have identified alterations in microbial composition in IBD patients. 2) Metabolomics profile changes in the metabolic output of hosts and microbes, including short-chain fatty acids and bile acids, both of which have been linked to IBD disease severity. 3) Genomic sequencing has facilitated the identification genes that influence the development of IBD such as mutations in NOD1/2 and IL-10 inflammatory pathways. 4) Metatranscriptomics facilitates the identification of transcriptomes as a proxy for functional output differences in IBD patients. 5) Metaproteomics characterizes changes in host and microbial stool- proteins affected by IBD with the additional ability to characterize post-translational modifications.

Stool has become an essential tool for noninvasive longitudinal characterization of IBD because it contains host, microbe-, and diet-generated biomolecules that reflect a host’s biological state. Due largely to costs, early stool-based IBD-omics studies often leveraged a single-omic technology at sole timepoints. However, researchers recognized that the limitations of single-omic cohorts were their inability to capture the diverse array of biomolecules that influence IBD pathology and subsequently the ability to develop a cohesive systems-level biological understanding.^24^ With decreasing costs, increasing throughput, accessibility, and profiling depth, the use of -omics technologies including genomics, transcriptomics, proteomics, and metabolomics is primed to transform the understanding of complex and multi-factorial diseases such as IBD. Multi-omic studies are now being used globally to profile patients across multiple molecular dimensions, resulting in greater and more relevant connectivity between microbes, their host, and diet. Thus, implementation of this approach holds promise in elucidating comprehensive molecular network profiles that can inform IBD treatment and monitoring (Figure 1).

In this review, we highlight the impact of -omics on IBD research, advancements in the field of stool multi-omics, and how its use has enabled researchers to identify critical and novel host–microbe interactions underlying IBD pathology. We also describe issues hindering its current utility and adoption, how to address them, and how to establish multi-omics as a common tool in IBD research.

## Early efforts characterizing the role of microbes in IBD

Initial characterizations of IBD suggested that aberrant immune responses were driven largely by pathologic dysregulation of host processes that are further influenced by microbes.^25–28^ Perturbations of intestinal epithelium in IBD and the resulting leaky gut were hypothesized to be the result of intestinal infections, leading to treatment with antibiotics (ciprofloxacin, metronidazole, rifaximin, clarithromycin, and others).^29^ However, these treatments had only modest efficacy, with most therapeutic benefits seen among patients with active colonic disease.^29^ Despite the modest efficacy, rates of antibiotic usage among IBD patients remains high and has inevitably contributed to the rise of antibiotic-resistant microbial strains. Although these antibiotic-resistant strains now plague clinicians, they also sparked significant interest in understanding host–microbe interactions with greater granularity to develop more targeted therapeutics.^29^ With this greater knowledge and the emergence of novel identification methods, IBD researchers began to consider the contributions of facultatively pathogenic components of the microbiome (pathobionts) such as *Enterococcus* spp. and *Escherichia coli*.^25,30^ However, a limited ability to culture microbes isolated from the gut hindered initial attempts to fully characterize their connection to IBD, although this critique has been at least partially addressed.^31^ The advent of culture-independent identification technologies such as 16S rRNA gene amplicon (16S) sequencing,^32,33^ in conjunction with the ease of stool-based sample collection,^22^ gave researchers a significant boost in the ability to identify connections between taxa and pathology, including a largely reproducible shift in *Firmicutes:Bacteroidetes* phyla ratios in both treated and treatment-naïve IBD patients.^34,35^ Efforts led by the American Gut Project revealed that relatively healthy individuals had antibiotic-respondent metabolic outputs uniquely linked to the personalized microbial environments, further supporting the need for increased personalized microbial profiles.^22^ These findings were further confirmed in a subset of IBD patients through a meta-analysis of 16S sequencing that showed reproducible shifts in microbial abundances in *Bacteroidetes, Firmicutes*, and *Proteobacteria* with five distinct microbial network modules present and two being distinct in those individuals diagnosed with either UC or CD.^19^ Thus, the development of shotgun metagenomics extended the identification utility of 16S data by offering researchers greater resolution in reliable species- and strain-level identifications, along with the ability to predict functional microbial pathways. Using this technology, early efforts profiling IBD patients suggested that these pathways were enriched in genes for the utilization of host-generated substrates, away from dietary sources.^36^ This further solidified the necessity to leverage multiple technologies in tandem to profile IBD, as even the significant capabilities of metagenomics were unable to capture the totality of complex interactions taking place.

## The path toward stool-centric IBD multi-omics

### Early efforts in stool-based IBD multi-omics

As previously described, initial attempts to explain IBD’s heterogeneity often focused on profiling gastrointestinal tract microbial composition and metabolic alterations. Microbes are significant consumers, processors, and producers of biomolecules, including bile acids, fatty acids, and amino acids, and thus their potential role in modulating gut metabolites was intuited.^37^ DNA-based methods such as shotgun metagenomic sequencing or 16S sequencing, and untargeted metabolomics became common stool-based multi-omic pairings for probing microbe-metabolite crosstalk in IBD.^38–40^ Using this combination, researchers revealed critical host–microbe connections in IBD. For instance, CD patients who underwent gastrointestinal tract resections had decreased microbial diversity and increased bile acid levels compared to pre-surgery levels.^41^ *Klebsiella* spp., *Enterococcus faecium*, and *Escherichia coli* were among the most frequently reported taxa with altered levels in IBD. Indeed, reduction in phylogenetic diversity is a consistent finding among IBD patients, a trend extending to related biospecimen types such as lavages.^19^ Additionally, through network modeling and sequencing data integration, it is now understood that the simultaneous increase in one taxa and reduction of another provides an alternative approach to linking disease state and microbial influence.^19^

Due to the noted connection between microbes and metabolites to disease state, multiple studies have directly tested the effects of metabolite administration on IBD disease models and profiled the results using genomic and metabolomic techniques.^42,43^ Phosphatidylcholine (PC) and sphingomyelin (SM) are membrane-bound molecules with several canonical roles including cell signaling and apoptosis, and are common dietary components. Previous research suggests that PC and SM are processed by gut microbes.^44,45^ Given this evidence, the effect of PC or SM administration on the microbiome and metabolome was tested in an IBD mouse model (Dextran sulfate sodium, DSS).^42^ PC and SM administration partially ameliorated DSS-dependent changes, with both shared and unique shifts in metabolome and microbiome profiles compared to DSS-only treatment. Interestingly, compared to controls, PC increased the abundance of indolepyruvate while decreasing with SM administration. PC also had a greater gross positive effect on host tissue compared to SM. Through multi-omic integration, significant correlations between *Lactobacillus* and indolepyruvate were identified. These correlations have also been observed independent of multi-omics, supporting the findings' validity; however, those studies did not benefit from multi-omic’s inherent ability to identify other network interactors.^46^ Among other noted roles, indolepyruvate is a potent aryl hydrocarbon receptor (AHR) agonist that modulates gut-homing immune cell activation.^47^ This connection suggests DSS-dependent loss of *Lactobacillus sp*., directly or indirectly decreases indolepyruvate levels, leading to decreased AHR activation and increased gut inflammation, and that PC administration corrects these imbalances to a greater degree than SM. While this hypothesis remains to be confirmed, it supports the notion that integrated-omics studies hold potential for uncovering cryptic host–microbe–diet connections.

Given these successes, it was unsurprising researchers began to include profiling approaches that help understand microbe-metabolite connections to the host’s transcriptional landscape. Using a combination of 16S and biopsy transcriptomics, Hernández-Rocha et al. identified a depletion of bile acid processing microbial genes in inflamed ileal mucosa of CD patients compared to non-inflamed tissue.^48^ Using Bayesian network analysis, they found that one of these predicted microbial genes, *baiCD*, was likely altering host levels of Angiopoietin-like 4 transcripts. Given angiopoietin-4ʹs role in wound healing and permeability, the loss of microbes responsible for these bile acid-responsive networks may have a direct link to host intestinal permeability. Moreover, it highlights the utility of leveraging complementary technologies for profiling host and microbial processes together in order to gain a greater understanding of the host–microbe cross-talk. In an *ex vivo* setting, this same-omics combination has been used to begin answering the question as to what factor contributes more to IBD’s inflammatory state. In an ingenious study design, Arnauts et al. revealed microbes derived from UC patients had a greater ability to decrease intestinal epithelial integrity compared to microbes derived from healthy donors despite the presence of inflammatory cytokines in both conditions.^49^ This suggests microbial processes are more likely to negatively affect host barrier functions as opposed to the general inflammatory cytokine milieu. Supporting these results, this work also found transcriptional markers of stress in epithelial tissue at significantly greater levels when exposed to microbes derived from UC patients compared to those derived from controls, regardless of epithelial tissue source (UC- or control-derived epithelium). In sum, these studies strongly suggest that profiling both the host and the microbial aspects of IBD is critical, further supporting the utility of multi-omics.

While understanding the molecular underpinnings of IBD remains central to most research efforts, significant efforts also focus on developing treatments. Fecal microbiota transplantation (FMT) is among the most promising of these treatments. These efforts were pioneered in cases of treatment-resistant *Clostridium difficile* infections but have recently demonstrated clinical efficacy in UC and cancer treatment.^50,51^ As FMTs gain popularity as effective treatments, it is critical that we comprehensively profile donor stool to understand what constitutes effective FMT sample for specific patients, and which samples may pose risks. In line with this, a recent meta-analysis of adverse FMT reactions over a 20-year span in 4,241 patients who underwent FMT, 19% had side effects including diarrhea and abdominal discomfort.^52^ In order to understand what features were associated with IBD disease modulation, a recent study combined shotgun metagenomics and untargeted metabolomics and noted that UC patient or healthy control donor class significantly influenced DSS model outcomes, with differential impacts on metabolic profiles along with associations with specific microbes.^53^ In healthy transplant mice, *Alistipes* and *Bifidobacterium sp*. and several anti-inflammatory metabolites, including the microbial-produced indoleacetic acid, were strongly correlated, suggesting that microbial products impact host immune tone. In contrast, UC patients with active inflammation were enriched in *Bacteroides sp*. and had associated with increased levels of amino acids, benzoic acids, and phenols, in line with previous evidence.^54^ However, the mechanistic connection between specific *Bacteroides sp*. and metabolites in the context of IBD remains largely uncharacterized. However, the negative outcome for mice receiving UC stool confirms multi-omic profiling allows for the identification of negative selection markers for FMTs. More direct evidence of this phenomenon from UC patients receiving FMTs demonstrated that microbial and metabolic profiles (228 identified metabolites) of patients post-transplant were differentiable between responder and non-responder patients.^55^ This correlated with altered pathways seen via shotgun metagenomic data involving metabolites such as lysine and heme, which have previously been shown to be associated with microbial pathogenesis and host inflammatory responses.^55,56^ These studies provide further evidence for the utility of multi-omic studies in identifying potential pathways of interest in IBD.

Despite the general success of the previous studies, deep integration of metagenomic and metabolomic data beyond correlation has represented a significant bioinformatic hurdle, and is applicable to the field of multi-omics in general. One solution proposed by Morton et al. addressed this shortcoming by leveraging simplified single-layer neural networks to generate conditional probability-based co-occurrence networks between metabolites and microbes.^57^ Using their tool to profile IBD patients, they reinforced many prior findings, including a striking co-occurrence of *Klebsiella* and bile acids, with more statistical rigor than previous associations. This co-occurrence may be due to the capability of *Klebsiella* to expand in environments unfavorable to other bacteria or point toward its ability to process primary bile acids, and thus thrive in altered bile acid level conditions. Indeed, multiple studies have shown that *Klebsiella* is resistant to several antibiotics and is consistently isolated from IBD patient stool.^58–60^ This represents a significant shift in analysis potential, moving beyond traditional correlations and toward a more direct causal connection.

### Stool metaproteomics: a powerful tool for characterizing host–microbiome interactions in IBD

Of the -omics previously applied to IBD, metaproteomics is still largely novel yet holds great potential. This is due to its ability to survey large search spaces consisting of microbial, host, and dietary proteins in a direct and highly quantitative manner using a relatively unbiased single assay. More fundamentally, its canonical place in the central dogma of molecular biology also makes it suitable for integration with transcriptional and genomic information compared to technologies like metabolomics whose products have the potential to be produced by multiple taxa. Prior studies have shown standard clinical markers of gut disease can be readily tracked using stool metaproteomics with high fidelity.^61^ Unlike genomics, metaproteomics can identify critical post-translational modifications and proteolytic cleavage patterns that play critical parts in IBD etiology and which cannot be determined by metatranscriptomics alone. Corroborating these claims, Li et al., showed that out of 30% of bacterial proteins identified in lavage samples from IBD patients 48% belonged to the phylum *Bacteroidetes* which supports metagenomics.^19,62^ Additional network analysis of proteomic data identified important protein classes specific to UC or CD – an important finding in biomarker assay development for disease categorization.^62^ Supporting the utility of metaproteomics, paired metaproteomic and metagenomic data from patient-matched stool samples showed minimal taxonomic and functional correlations (*ρ* = 0.31, 0.14 respectively).^61^ Because proteins are the final stage of the central dogma, this evidence suggests it is crucial to have multiple streams of data covering the same domains. Other groups have shown that metaproteomics (compared to other -omics technologies) provided more complete holobiont profiling, even when the initial protein isolation methods were selective.^63^ Despite this promise, based on current usage, metaproteomics still represents a novel inclusion in multi-omic studies despite significant utility for studying IBD.^64–66^ Herein, Table 1 presents key information that contributed to the success of recent multi-omic works with the integration of metaproteomics.

**Table 1.** Overview of meta-proteomic approaches used in multi-omic datasets of IBD-related studies.

Study	Quantification Method	N	Fractionation Method	Protein IDs	Reference Metagenomic datasets for Protein ID
Lloyd-Price et al., Nat. 2019	Label-free	132	High pH Reversed-phase fractionation	<10,000	Human Microbiome Project Dac web portal (https://www.hmpdacc.org/ihmp)
Mills & Dulai et al., Nat Microbiol. 2021	Tandem Mass Tag (TMT) labeling• TMT10	Cohort 1: 40Validation Cohort 2: 210	Basic pH Reverse-phase liquidchromatography	Cohort 1:<50,000Validation Cohort 2:<100,000	Integrated generalized human microbiome reference genome (Li 2014).
Wastyk et al., Cell. 2021	Tandem Mass Tag (TMT) labeling• TMT11	36	None	<10,000	Uniprot Swiss-ProtHomo sapiens(taxon ID 9606), the Human Micro-biome Project (FASTA file downloaded from https://www.hmpdacc.org/hmp/HMRGD/), and in house curated database.

IBD studies using metaproteomics as part of their multi-omic framework have revealed critical commonalities that were previously untethered from larger -omic frameworks. One consistent finding involves the species *Bacteroides vulgatus* and its inherent relationship to IBD. Recently, several groups confirmed this connection in UC patients to varying degrees.^53,65,67^ Using a stool-based multi-omic workflow, the studies identified *B. vulgatus* as a major contributor of disease severity for UC patients. Moreover, one study among these revealed metaproteomic data predicted disease severity to a greater degree than its metagenomic counterpart.^65^ Metaproteomics further extended this association by identifying that secreted *B. vulgatus* proteases were strong drivers of the disease severity connection, a finding that was confirmed using UC mouse models and protease inhibitors. This multi-omic study provided critical insights into the mechanisms driving disease severity that were previously unknown, in part due to increases in feature identifications and proteome coverage identified key increases in stool and serum protease levels. This in turn led to the testing and validation of their findings in animal models, suggesting multi-omics can generate testable hypotheses that reflect the underlying biology with great accuracy. Moreover, the use of isobaric-tandem-mass tag (TMT) technology has led to a major increase in proteome coverage compared to traditional label-free quantifications (Table 1). The use of TMTs with the implementation of the Orbitrap mass analyzers has both facilitated high-throughput studies by reducing instrument time and increased the total number of quantifiable proteins, allowing for a greater than 20-fold increase in coverage of the gut metaproteome. In sum, metaproteomics of stool holds significant promise given its powerful integrative potential and ability to deliver functional information that can help support other omics technologies such as metagenomics.

## The rise of large-scale microbial multi-omics studies

Due to historically high costs and lack of access, a majority of stool-based multi-omic IBD studies have focused on leveraging at most two methods simultaneously, most commonly metagenomics and metabolomics. To date, few IBD-focused studies have combined more than three -omics data streams. However, the field now understands the need to utilize a multi-omics strategy with more than two profiling technologies to capture IBD’s heterogeneous phenotypes. Indeed, Lloyd-Price et al.’s staggering effort collected longitudinal stool-based-omics data (in addition to other non-stool-based-omics) from a large cohort of IBD patients.^68^ Deep integration of these data yielded an association network with over 51,000 connections, a large subset of which were highly significant. Heavily connected networks revealed associations between *Faecalibacterium prausnitzii*, cholesterol, and inosine regulatory networks; both of which were largely downregulated during IBD-related dysbiosis. Recapitulating prior work, they also revealed microbe-bile acid networks, many of which have been previously characterized as arising in conditions of increased bile acid presence. Interestingly, few host features, including well-known markers such as calprotectin formed major hubs, and metaproteomic features were largely missing from enrichment networks, possibly due to the small feature set compared to other metaproteome studies.^65,66^ In contrast, metaproteomics played a dominant role in a large multi-omic effort focused on UC.^65^ This study also revealed the intricate connectivity and utility of each data type for both hypothesis generation and validation. Metaproteomic protein abundance displayed stronger correlations to disease severity than metagenomic data generated from the same patients. While the bioinformatic rationale behind the finding remains to be tested, it is possibly due to A) a general lack of correlation between metagenomic counts and actual expression or B) a decrease in strain-level ‘signal splitting’ that may occur to a greater degree in metagenomic data compared to metaproteomic data when multiple strains of the same species are present in databases. Indeed, a recent metagenomic survey of samples using a general and strain-level database identified over multiple strains of *Akkermansia muciniphila*, a well-known host mucus metabolizer, suggesting strain diversity may be complicating species-level analyses of metagenomic data.^7^ This multi-omic effort uncover the molecular effects hyperbaric oxygen therapy (HBOT) has on UC patients using a combination of stool- and tissue-based-omics.^7^ Pathologically low levels of oxygen are present in UC patients, which HBOT corrected and resulted in improved clinical and endoscopic disease activity. However, due to the increased oxygen levels, researchers suspected an effect on both host and microbial composition. Patients failing to respond to HBOT had unchanged levels of mucus pre- and post-treatment (confirmed in both stool and biopsies using proteomics). In tandem, metagenomics identified levels of *A. muciniphila*, were reduced in responders but not in non-responders, despite this being an obligate anaerobe and all patients receiving extremely high levels of oxygen to the colon. Further strain-level analysis confirmed *A. muciniphila* strains present in non-responders were consistent with recently identified aerotolerant strains while responder strains did not, negating HBOT’s beneficial effects. Whether *A. muciniphila* directly alters the inflammatory state, or its mucus-clearing propensity allows more pathological microbes to influence intestinal epithelium remains to be seen, however the results serve as a test case for the use of large multi-omics to drive future mechanistic follow up studies. Currently few studies have used these large multi-omic approaches. However, this is expected to soon change as the clear advantages in its implementation continue to be highlighted. Importantly, it is an approach that will impact the understanding of other complex diseases.

When treatment modalities and efficacy are being assessed, implementation of a multi-omic approach has also shown promise. A recent study led by Lee et al., revealed paired stool and blood multi-omic analyses of 189 IBD patients yielded highly predictive biomarkers of therapeutic success.^69^ The study collected samples from patients prior to immuno-therapy, during (14 weeks), and post-remission (week 52), which facilitated the identification of important microbial shifts in metagenomic feature sets. This work also subcategorized individuals who responded to treatment and reached a state of remission and identified proteins positively associated with remission states in IBD patients, such as CASP8. They went on to test whether these parameters could predict therapeutic response in 21 participants with available multi-omic data and saw an impressive predictive response of 96.3%. Further, the addition of -omic datasets to clinical features previously used in isolation to predict remission was significantly improved suggesting future clinical studies aiming at prediction would be well served by integration of multi-omics features.^69^

## The future of IBD-omics: connecting host–microbe interactions to diet

Current evidence suggests environmental factors including diet continue to be identified as a component contributing to IBD, however the degree to which it significantly influences disease onset or exacerbates symptoms is not well characterized. Major sequencing efforts suggest that diet shifts gut microbial composition in both humans and rodent models with similar ratios of bacterial phyla with *Firmicutes, Bacteroidetes*, and *Proteobacteria* dominating composition.^1,70,71^ In the context of IBD, diet’s ability to impact microbial diversity significantly impacts gut dysbiosis.^17,72–74^ While early work often leveraged genomic technologies, these findings have since been correlated to protein levels as well.^75^ Despite these efforts, the complexity of the microbiome, when coupled to individual habits and environment,^1^ makes it difficult for IBD studies to adequately control for diet regimens. This leads to the following questions: can diet prevent or treat IBD? Or can the microbial shifts, largely influenced by diet and host factors, help identify targeted treatments leading to greater remission rates?

These questions are broad and require significant efforts to answer, however they represent a starting point for more pointed questions using multi-omics. Despite its potential utility for elucidating the role of dietary components in IBD, both single- and multi-omic studies are still rare. Nonetheless, -omics studies are now beginning to impact our understanding of diet’s contribution to dysbiosis. This is especially true for the case of food industrialization and western diets.^22,73,76^ Montrose et al. leveraged several -omics including metatranscriptomics, metabolomics, and 16S sequencing to elucidate the effect of high-fructose diets, a common component of western diets, on a DSS model of intestinal inflammation.^77^ In line with similar studies, they showed that high fructose diets altered the microbiome resulting in increased DSS-induced colitis scores, and that antibiotic administration ameliorated the enhancing effect a high fructose diet had on this colitis model. Metabolomic and metatranscriptomic data revealed a decrease in the production of bile salt hydrolases, echoing previous studies and further highlighting the critical role bile acids play in host physiology and microbial metabolic output. In a similar study, Lin et al. recently combined metabolomics and transcriptomics to profile changes in mice fed a western diet.^78^ These mice displayed increased colitis compared to those fed normal chow, as shown through a reduction in colon length, increased immune cell infiltration, upregulation of gastric cancer-associated genes, and decreased production of metabolites such as kynurenic acid.^78^ Kynurenic acid (acquired through the metabolism of dietary tryptophan) is increased in patients with type 2 diabetes and IBD, however, its role as an inflammation modulator is disputed.^79,80^ This may be due to its importance to multiple organs such as the brain, liver, and its endocrine function despite rodent models revealing its concentrations increase sequentially from the proximal, middle, and distal ileum.^81,82^ However, transcriptomics data generated using a TNBS-based colitis model revealed that among differentially expressed genes related to the production of proteoglycans involved in cancer were among the most altered.^78^ Proteoglycans have acquired an increased interest as they function as a secondary barrier to multiple cell types, including endothelial and immune cells – both cell types with critical roles in IBD-pathology.^83^ Indeed, assessment of Heparan sulfate proteoglycans (HSPs), known to be associated with inflammatory responses as those seen in IBD in the rodent model interleukin 10 knockout (IL10-/-) showed reduction of HSP, syndecan-1.^83^ Although Lin and group integrated metabolomics and transcriptomics that corroborated with previous functional studies, defining mechanisms connecting diet-based barrier disruption lacked. This is one of the limitations that multi-omic integration faces when only selecting two -omic datasets. Transcriptomic data, although powerful, is still largely predictive as the presence of post-translational modifications determines functional protein state, which in turn may impact disease phenotypes. This suggests single-omics can be useful for certain genetically driven diseases, but a multi-omic approach may be more useful for answering multi-factorial questions such as those relating to diet-metabolite-microbe interactions in IBD.

From a therapeutic perspective, profiling diet’s influence on host–microbe interactions has resulted in IBD-specific diets designed to reduce inflammation.^84^ However, untangling diet’s influence on both host and microbes in a state of IBD at the molecular level remains a challenge. Extant IBD-omic studies that have focused on diet have demonstrated its impact on microbial composition.^85,86^ These studies suggest that dietary components influence microbial composition and modify a host’s risk for cancer, diabetes, and IBD.^73,76,85–87^ In the case of risks associated with diet and IBD, Patterson et al. assessed the effects of a western-style diet (WSD) on colitis severity in the IL10-/- colitis mouse model.^83^ Physiological data revealed that pre-exposure to a WSD exacerbated colonic injury upon chemical induction of colitis with TNBS as shown through colonic length and colonic histopathological scoring.^83^ In the case of specified diet treatments and interventions, their effectiveness remains highly variable suggesting underlying factors such as microbial composition influence outcomes. For instance, diets containing components such as fiber, fermentable oligo-, di-, monosaccharides, and polyols (e.g., FODMAP) can aggravate symptoms and inflammatory conditions.^88,89^ As such, clinicians prescribe low-FODMAP diets, Mediterranean diets, and specific carbohydrates.^90^ These diets alleviate symptoms in a subset of patients by reducing the activity of pathways involved in secretion of fecal calprotectin, C-reactive protein, and immune cell recruitment.^89,91,92^ This is largely attributed to the three components targeted in the host–microbe interactions – short-chain fatty acids (SCFAs), bile acids (BA), and vitamin metabolism, all of which are altered by resident gut microbiota.^89,92^ It has been shown that SCFA production is largely derived from microbiota-accessible carbohydrates and is critical in maintaining barrier function and integrity, while BAs largely help regulate host metabolism, and vitamins derived from nutrient consumption largely aid in both barrier function and immune response – a key deregulation in IBD.^89,92^ Diets low in fats and high in fiber have shown a decrease in amyloid A, an increase in alpha diversity, with an accompanied improved quality of life in UC patients.^89,91,92^ However, the microbiome’s role in the efficaciousness of these modified diets remains to be comprehensively characterized.^90^ Indeed, the lack of a response in most patients strongly suggests an incomplete understanding of diet–microbe–host interactions.^90^ Meta-analyses of diet intervention trials in CD further support the notion that many studies were statistically underpowered, contained undefined study bias, and overall had limited clinical applicability, with highly variable patient outcomes.^93^ These inconsistencies hinder the ability of clinicians to confidently prescribe IBD-modifying diets. Given the utility multi-omic approaches have for better understanding diet–host–microbe interactions, their use in defining the role diet plays in IBD is warranted.

Supporting this notion, Wastyk et al. leveraged a multi-omic approach to identify high-dimensional differential responses to increased fiber or fermented food intake in human subjects.^94^ High-fiber diets did not impart changes to microbial diversity metrics, as measured using shotgun metagenomics. Here, protein abundance derived from microbes suggested a possible increase in microbe production or secretion functions.^65,94^ Interestingly, samples from participants on high-fiber diets showed varying responses with respect to circulating inflammatory responses, which correlated to fluctuations in microbial composition. Conversely, participants undergoing a high-fermentation diet showed a decrease in inflammatory markers such as IL-10 and IL-6, both cytokines shown to be implicated in IBD, suggesting it is possible that fiber type has a complex, hidden impact on specific microbes present in only a subset of patients.^94^ Thus, this study shows the potential multi-omics approaches have in elucidating previously unknown gut-centric interactions, which can be applied to IBD in future studies.

## Guidance and considerations for multi-omics studies

Evidence presented thus far has highlighted how advanced technology and bioinformatic-led data integration has facilitated our understanding of the molecular basis of IBD and the host–microbe–diet axis. However, the inherent complexity of each technology has thwarted many attempts to deeply integrate multi-omic feature sets, yielding minimal additional information. Here, we provide general considerations designed to help avoid common multi-omic study pitfalls. While we avoid in-depth technical recommendations about specific -omics processing, as they are beyond the scope of this review, we include a table of useful studies that can provide guidance and useful protocols (Table 2).

**Table 2.** List of IBD-related studies with information relating to -omics used and novel information gathered from data integration.

Study	16S rRNA	Shotgun Meta genomics	Transcriptomics	Metabolomics	Metaproteomics	Novel Information Gathered
Lloyd-Price et al., Nat. 2019	Included	Included	Included	Included	Included	*Identified distinct dysbiotic event differences between UC and CD that largely correlated to colonic epithelium molecular networks.*
Mills & Dulai et al., Nat Microbiol. 2021	Included	Included	Included	Included	Included	*Identified microbe-derived proteases correlated to ulcerative colitis (UC) as a function of disease severity.*
Wastyk et al., Cell. 2021	Included	Included		Included	Included	*Identified important host proteins and CAZyme relationships in response to diet interventions that showed improved inflammatory responses.*
Lin et al., J Inflamma Res. 2022			Included	Included		*Identified co-expression networks important for western diet’s contribution to increased colitis susceptibility.*

First, it is important to *a priori* establish goal(s) for multi-omics study to minimize time, effort, and resources, acquiring excessive or insufficient amounts of data. In this spirit, multi-omics often falls into two categories that are not mutually exclusive: exploratory or confirmatory. The former refers to novel comparisons where little information is available to guide mechanistic interrogations, while the latter is often more targeted in its -omics choices. Our anecdotal experience suggests that exploratory studies benefit most from multi-omic frameworks, as the extent to which any single-omics type discerns disease subtypes or treatment effects is not easily predicted. For example, our lab and others have previously shown that overall profile alterations in IBD can be minimally present in one feature set, while others reveal robust changes.^65,94^ In contrast, studies focused on validating specific findings often lend themselves to carefully selecting specific -omics, especially when time (e.g. manuscript resubmission) is critical. Nevertheless, confirmatory studies can still serve as jump-off points for future studies, and thus multi-omics can still be useful in these cases.

Next, we recommend compiling comprehensive cohort metadata, as previous efforts have detailed.^95^ Included in it, we suggest a shared subject identifier be applied to all -omic sets used, and as much categorical and quantitative data on it as possible. If the study involves clinicians, it is critical to involve them from the inception to collect all appropriate patient information. Indeed, comprehensive metadata can significantly impact the outcome of studies and serve to clarify the statistical feasibility of questions to be answered. In IBD, some valuable metadata fields are endoscopic disease activity scores (UCEIS, Mayo, SES-CD, etc.), prior surgeries, and historical/current treatment regimens. Surprisingly, a majority of IBD studies have limited endoscopic data, instead relying on symptom-based disease classification, which is known to be inaccurate in nearly 50% of patients.^96^ In addition, it is strongly recommended that healthy controls be included in large studies, as they can serve as initial conditions, and when possible longitudinal data to capture within-patient shifts in disease states, particularly during transitions in and out of disease remission. These guidelines can help avoid issues with statistical power in highly heterogeneous cohorts.^97,98^

The utility of multi-omics lies in its ability to generate cohesive feature sets built from disparate data types, and far too often samples are included in a study but are not profiled by all -omics due to processing issues or sample availability. As such, we suggest study subjects be filtered based on their ability to adequately provide biomaterial for all -omics being used. In general, it is also advisable to favor samples with excess material available in the event processing issues arise. Additional sample-centric concerns include controlling for sample collection and processing effects that may affect data integrity.^99,100^ To avoid this, we advise processing all samples for an -omics at a single time, preferably using high-throughput methods amenable to robotic processing while randomizing samples across batches. Where available, advanced quantitative labeling should be used to further mitigate batch effects, especially when acquiring metaproteomics data, as it significantly increases identifications and quantitative power. Selecting appropriate bioinformatic processing pipelines^99–101^ and analysis standards will also help avoid downstream Type I/II statistical errors.^98^

Finally, after all features are acquired, processed, and integrated, it is critical to ground studies by testing that features noted in prior studies are adequately recaptured. For instance, multi-omic IBD studies should confirm previously described trends such as shifts in taxon proportions are also present in their cohort. Here, healthy controls help tremendously. For instance, using stool-based metaproteomics, studies should note a robust increase in neutrophil-related proteins compared to healthy controls. Similarly, IBD patients generally exhibit altered bile acid levels as seen by untargeted metabolomics. In general, whatever confirmatory trends are decided upon should be confirmed, and if they are not observed, processing pipelines should be checked for errors. This initial quality control measure can significantly prevent wasted time and effort during downstream analysis.

## The promise and challenges of multi-omics, IBD, and precision medicine

The past decade has seen an explosive adoption of -omics technologies in virtually every field of research and their use is continuously increasing. Given the impact single-omics has had on acceleration of both discovery- and hypothesis-driven science, multi-omics represents the next logical adoption. While individual-omics have played a critical role in identifying the important microbes correlated with disease, the research we have highlighted strongly suggests that multi-omics is needed for identifying mechanisms driving these highly complex pathologies.

Moving into the future, we predict that decreasing price-per-sample and increasing accessibility will accelerate multi-omics usage for IBD studies. Consequently, we believe cohort sizes will continue to expand, allowing more comprehensive profiling of IBD subtypes (e.g., factors differentiating ileal and colonic CD) and further enabling the study of complex interactions networks such as diet and IBD. Given its prominence in several recent studies, we also predict the field of metaproteomics will play a more prominent role in IBD research. Historically, proteome preparation pipelines were significantly more laborious than their nucleic acid counterparts. However, recent advancements in multiplexing (TMT, iTRAQ, etc.) and processing have at least partially addressed this.^64^ Similarly, proteome spectral identification software has historically struggled to identify more than a few hundred proteins from stool. However, methods now exist that allow upward of 100,000 quantified host and microbial proteins, with room for even greater numbers of identifications as mass spectrometer technology and accompanying software continue to advance.^65^

Despite significant potential, large-scale multi-omics studies (using at least four-omics on a single biospecimen) in IBD research, or any field, are still largely novel. For mass adoption of multi-omics to become reality, several issues in the field must be addressed. One often overlooked critical issue for stool-based multi-omics is a lack of large (>1000 individuals) benchmarked cohorts that accurately approximate the range of features in a healthy cohort. Solving this issue is hindered by the significant costs and data acquisition time, suggesting federal-level efforts mirroring the human genome project may be required, despite the ongoing academic efforts.^23,102^ Next, each stool-based-omics has several potential processing pipelines, data normalization techniques, and imputation standards employed to generate a final feature table. This variation increases statistical complexity and decreases consistency in results. While many efforts that address properly merging feature sets are described, a majority focus on integration of multi-omics profiling a single organism, which is not reflective of stool-based multi-omic efforts.^103,104^ Therefore, a common bioinformatic pipeline that generates consistent and lab-to-lab reproducible results, regardless of initial processing pipeline, would represent a major milestone for both general biological understanding and clinical outcomes. An additional hurdle is a lack of standardized metadata collection practices in IBD-omics studies, where study-to-study sample metadata practices limit meta-analyses. While some minimal metadata standards have been established for the inclusion of data into public repositories (both raw data and processed) such as Qiita,^105^ MassIVE,^106^ EBI-ENA, NCBI, and ProteomeXchange,^107^ there is a critical need for a ‘cross-cohort identifier’ shared between repositories.^105,107^ Given the difficulties recruiting IBD patients for lengthy studies, a cross-cohort-identifier would further serve to link subjects to all included -omics sets in a study and enable more seamless inter-cohort comparisons.

While these issues largely reflect a lack of standardized statistical practices, additional issues exist that require more field-wide efforts. For instance, we foresee the inclusion of dietary components as a major contributor to disentangling the current study-to-study variability. However, current food-based metadata and diaries are often entered as generalized inputs (e.g., protein bar, chicken soup, burrito, etc.). We believe increased granularity is necessary, especially for the inclusion of appropriate databases, which can limit spurious identifications due to sequence or mass homology. This will likely require a significant effort in profiling of individual diet components, which to date has not been undertaken. However, efforts to develop relational ‘food trees’ have helped inform these efforts.^108^ Multi-omic progress is also impeded by the tremendous percentage of unannotated features, sometimes referred to as the ‘omics dark matter’. Further, zero-inflated matrices generated by current -omics technology is a major source of variation. These two factors together can lead to largely meaningless correlations and significantly mitigate the utility of multi-omics. Given a majority of proteomics and even metabolomics is inferred by genetic sequences, targeted efforts at deep sequencing microbes with significant numbers of unannotated genes should be undertaken. Several additional largely microbe-centric problems also plague multi-omic studies also exist, such as an inability to assign certain sequences.

Regardless, the issues listed above are not intractable and thus IBD research is primed for an explosion of stool-based multi-omics research. With the continued rise in idiopathic diseases like IBD, the need for precision-medicine-driven solutions is critical. Toward this goal, we envision stool-based multi-omics will significantly expand our mechanistic knowledge of diet-microbe-host interactions that influence IBD pathology and bring about true precision medicine treatments.
